# Functional Characterization of Breast Milk-Derived *Lactobacillus paragasseri* HUH02 Postbiotic Preparations with Immunomodulatory, Skin Barrier-Protective, and Anti-Melanogenic Activities

**DOI:** 10.3390/microorganisms14081777

**Published:** 2026-08-12

**Authors:** Ran Lee, Hyun-Chul Park, Chul-Sung Huh, Hyunjoon Park, Hyeon-Woo Sim, Joo-Sang Lee, Jun-Chul Kang, Hyun-Jung Park

**Affiliations:** 1 Department of Biotechnology, College of Biomedical & Health Science, Konkuk University, Chungju 27475, Republic of Korea; ranran2424@gmail.com; 2 Department of Cosmetic Industry, Graduate School, Chungbuk National University, 194-21, Cheongju 26844, Republic of Korea; seung-hee@hanmail.net; 3 Graduate School of International Agricultural Technology, Seoul National University, Pyeongchang 25354, Republic of Korea; cshuh@snu.ac.kr; 4 Biodome Co., Wonju 26365, Republic of Korea; 5 Institute for Liver and Digestive Diseases, Hallym University, Chuncheon 24252, Republic of Korea; 6 Department of Animal Biotechnology, College of Life Science, Sangji University, Wonju 26339, Republic of Korea; opensesim@gmail.com; 7 R&D Center, Dermalab Co., Ltd., #510 Ace Gwanggyo Tower 1, 17, Daehak 4-ro, Yeongtong-gu, Suwon-si 16226, Republic of Korea; rnd_01@dermalab.co.kr (J.-S.L.); rnd_11@dermalab.co.kr (J.-C.K.)

**Keywords:** *Lactobacillus paragasseri* HUH02, postbiotics, immunomodulatory activity, epithelial barrier enhancement, antimelanogenic effects

## Abstract

Breast milk-derived lactic acid bacteria (LAB) are promising sources of functional postbiotics. In this study, we evaluated the immunomodulatory, epithelial barrier-enhancing, and anti-melanogenic properties of nonviable HUH02 preparations, including heat-killed cells (HUH02-HK) and microfluidized preparations generated at 20,000 psi (HUH02-2psi) and 40,000 psi (HUH02-4psi), derived from the breast milk-derived *Lactobacillus paragasseri* HUH02 (*L. paragasseri* HUH02). HUH02 derivatives were evaluated in RAW264.7 macrophages, HaCaT keratinocytes, and B16F1 melanoma cells. All exhibited low cytotoxicity at functional concentrations. In Lipopolysaccharide (LPS)-stimulated RAW264.7 macrophages, HUH02 derivatives markedly reduced nitric oxide production and suppressed the expression of inflammatory mediators, restored macrophage morphology toward a non-activated phenotype, and reduced iNOS protein expression. In HaCaT keratinocytes, HUH02 derivatives enhanced wound closure and the expression of epithelial barrier-associated genes, including *ZO-1* and *Occludin*. Moreover, microfluidized derivatives, particularly HUH02-2psi and HUH02-4psi, significantly enhanced transepithelial electrical resistance, indicating improved epithelial barrier integrity. In B16F1 melanoma cells, HUH02-derived preparations exhibited antimelanogenic effects by reducing intracellular melanin accumulation and suppressing the expression of melanogenesis-related genes and proteins, including *Tyr*, *TRP-1*, *TRP-2*, and MITF. HUH02 was identified as *L. paragasseri* based on 16S rRNA, average nucleotide identity, and whole-genome phylogenetic analyses. Genome-based safety assessment revealed the absence of antibiotic resistance genes, virulence factors, plasmid replicons, and pathogenicity-associated markers. These findings demonstrated that breast milk-derived *L. paragasseri* HUH02 postbiotics exhibit multifunctional biological activities, including immunomodulatory, epithelial barrier-enhancing, and antimelanogenic effects.

## 1. Introduction

The skin is the largest organ in the human body and is continuously exposed to environmental stressors. It serves as an essential physical and immunological barrier that protects the body against microbial invasion, ultraviolet radiation, oxidative stress, and other environmental insults [1]. Structurally, the skin consists of three major layers: the epidermis, dermis, and subcutaneous tissue. Among these, the epidermis functions as the primary defensive barrier against external stimuli and pathogens [2]. Keratinocytes account for approximately 90% of epidermal cells and play essential roles in maintaining epidermal homeostasis and promoting wound healing through epithelial regeneration [2,3]. Therefore, enhancing keratinocyte function is an important strategy for preserving skin integrity and improving skin health [4]. Because the epidermis is constantly challenged by environmental factors, considerable efforts have been devoted to identifying bioactive substances that enhance skin barrier function and accelerate tissue repair [5].

Microbially derived compounds have attracted increasing attention because of their diverse biological activities and potential dermatological applications [6]. In addition to keratinocytes, melanocytes located in the basal epidermis regulate skin pigmentation through melanin synthesis and transfer, thereby contributing to skin homeostasis [7,8,9]. Lactic acid bacteria (LAB) have a long history of safe use and well-established health-promoting properties. Although LAB are primarily recognized for their gastrointestinal benefits, accumulating evidence indicates that they can also improve skin health by supporting epidermal function, maintaining skin homeostasis, and protecting against environmental stress [6,10]. Recent studies have further demonstrated that LAB-derived probiotics and postbiotics can improve skin barrier integrity, regulate cutaneous immune responses, alleviate oxidative stress, and modulate melanogenesis, highlighting their potential applications in cosmetic and dermatological products [6,11,12].

Recently, interest has shifted from live probiotics toward postbiotics, defined as preparations of inanimate microorganisms and/or their components that confer health benefits to the host [13]. Compared with live microorganisms, postbiotics offer advantages including greater storage stability, improved formulation flexibility, and reduced safety concerns. Consequently, heat-killed bacteria, bacterial lysates, and cell-derived fractions have emerged as attractive ingredients for cosmetic and functional food applications [14,15]. Importantly, the biological activities of postbiotics depend not only on the bacterial species and strain but also on the processing method. Mechanical disruption techniques, such as high-pressure homogenization, can alter bacterial structures and facilitate the release or increased accessibility of intracellular bioactive compounds. These changes may enhance the interaction of bioactive molecules with target cells [1]. Nevertheless, comparative studies evaluating the skin-related biological activities of heat-treated and mechanically disrupted postbiotic preparations remain limited [1].

Breast milk is an important reservoir of beneficial microorganisms that contribute to neonatal microbial colonization and immune development [16]. Breast milk-derived *Lactobacillus* strains are of particular interest because they originate from a host-adapted environment and possess physiological characteristics that may promote host–microbe interactions [17,18]. However, despite increasing interest in breast milk-derived probiotics, their potential applications in skin health have received comparatively little investigation. Furthermore, although several *Lactobacillus paragasseri (L. paragasseri*) strains have been reported, the biological properties of individual strains are highly strain-specific. HUH02 is a breast milk-derived *L. paragasseri* strain isolated and cultured by our research group and is not proposed as a novel taxonomic strain. To the best of our knowledge, no previous studies have reported the biological or functional characteristics of this specific strain. Therefore, this study investigated the skin-related biological activities of the breast milk-derived *L. paragasseri* HUH02 isolate. We performed a comprehensive functional characterization of HUH02 by evaluating its antioxidant, anti-inflammatory, antimicrobial, skin barrier-enhancing, wound-healing, and anti-melanogenic activities. To determine the influence of processing methods on its biological properties, heat-killed HUH02 and preparations mechanically disrupted by high-pressure homogenization at 20,000 psi and 40,000 psi were compared using RAW264.7 macrophages, HaCaT keratinocytes, and B16F1 melanoma cells. Through these analyses, we aimed to determine whether processing-dependent structural modification of HUH02 alters its biological activities and to provide fundamental evidence supporting the potential application of HUH02-derived postbiotics as multifunctional ingredients for skin health.

## 2. Materials and Methods

### 2.1. Preparation of Heat-Killed and Microfluidized HUH02

*L. paragasseri* HUH02, provided by Biodome (Wonju, Republic of Korea), were cultured aerobically in animal component-free MRS medium (Kisan Bio, Seoul, Republic of Korea) at 37 °C. Before experimentation, the cells were subcultured twice for 18–24 h. The cultured cells were harvested through centrifugation at 3000 rpm for 20 min, and the resulting cell pellet was resuspended in sterile distilled water and concentrated 10-fold to obtain a final bacterial suspension of approximately 2.8 × 10^9^ CFU/mL. Heat-killed HUH02 cells (HUH02-HK) were prepared by incubating the bacterial suspension in a water bath at 100 °C for 30 min. Microfluidized HUH02 preparations were generated using a Microfluidizer (MN400BF; Micronox, Seongnam, Republic of Korea). The bacterial suspension underwent high-pressure disruption five times at either 20,000 psi (HUH02-2psi) or 40,000 psi (HUH02-4psi). The morphological characteristics of live HUH02 cells, heat-killed cells, and microfluidized preparations were examined at the Korea Research Institute of Bioscience and Biotechnology (KRIBB, Daejeon, Republic of Korea) using field-emission scanning electron microscopy (FE-SEM).

### 2.2. Scanning Electron Microscopy (SEM) Analysis

For SEM analysis, bacterial samples were fixed for 4 h in a solution containing 2.5% paraformaldehyde and glutaraldehyde in 0.1 M phosphate buffer (pH 7.2). The fixed samples were postfixed with 1% osmium tetroxide prepared in the same buffer for 1 h, followed by dehydration through a graded ethanol series. The dehydrated samples were then substituted with isoamyl acetate, dried using a CO_2_ critical point dryer, and coated with gold using a sputter coater (SMC-10S; SEMIAN, Seoul, Republic of Korea). The samples were examined using a field-emission scanning electron microscope (FEI Quanta 250 FEG; Thermo Fisher Scientific, Waltham, MA, USA) at KRIBB.

### 2.3. Cell Culture

The murine macrophage cell line RAW 264.7, human keratinocyte cell line HaCaT, and murine melanoma cell line B16F1 were obtained from the Korea Cell Line Bank (KCLB, Seoul, Republic of Korea). The cells were passaged twice before the experiments to ensure stable growth. All cell lines were cultured in Dulbecco’s Modified Eagle’s Medium (DMEM; SPL, Pocheon, Republic of Korea) supplemented with 10% heat-inactivated fetal bovine serum (FBS; Gibco, Grand Island, NY, USA) and 1% antibiotic−antifungal solution (Gibco). Cells were maintained at 37 °C in a humidified environment containing 5% CO_2_, and the culture medium was replaced every two days. RAW 264.7 cells (passages 10–15), HaCaT cells (passages 20–26), and B16F1 cells (passages 25–32) were used for all in vitro culture experiments.

### 2.4. Cell Viability Assay

Cell viability was analyzed for all three cell types. RAW 264.7, HaCaT, and B16F1 cells were seeded into 48-well plates at a density of 3 × 10^4^ cells/well and cultured for 24 h to allow cell attachment. Subsequently, the cells were treated with heat-killed HUH02 (HUH02-HK), HUH02-2psi, or HUH02-4psi at concentrations of 10^4^–10^8^ CFU/mL for 24 h. After treatment, EZ-Cytox solution (10%, *v*/*v*; DoGenBio, Seoul, Republic of Korea) was added according to the manufacturer’s instructions, and the cells were incubated at 37 °C for 1 h. Absorbance was measured at 450 nm using a microplate reader (Epoch; BioTek Instruments, Winooski, VT, USA). Cell viability was calculated as a percentage relative to the untreated control. HUH02-derived preparations were added at bacterial cell equivalent concentrations of 1 × 10^4^, 1 × 10^5^, 2 × 10^5^, 5 × 10^5^, 1 × 10^6^, 1 × 10^7^, and 1 × 10^8^ CFU/mL, corresponding to approximate bacteria-to-cell ratios of 0.33:1, 3.3:1, 6.7:1, 16.7:1, 33:1, 333:1, and 3333:1, respectively. As the HUH02 preparations were nonviable (heat-killed or microfluidized), bacterial cell equivalents rather than multiplicity of infection (MOI) were used to describe the treatment conditions.

### 2.5. Nitric Oxide (NO) Production Assay

Nitric oxide production in RAW 264.7 macrophages was measured using the NO Plus detection kit (iNtRON Biotechnology, Seongnam, Republic of Korea) according to the manufacturer’s instructions. RAW 264.7 cells were seeded into 48-well plates at a density of 3 × 10^4^ cells/well and cultured for 24 h at 37 °C in a humidified incubator with 5% CO. The cells were pretreated with lipopolysaccharide (LPS; 1 μg/mL) for 1 h and then treated with HUH02-HK, HUH02-2psi, or HUH02-4psi at concentrations of 1 × 10^6^, 1 × 10^7^, and 1 × 10^8^ CFU/cm^2^, respectively. Dexamethasone (DEX; 1 μM) was used as a positive control. After 24 h, the culture supernatants were collected and centrifuged at 10,000× *g* to remove cell debris. Equal volumes (100 μL each) of supernatant and Griess reagent were mixed, and absorbance was measured at 540 nm using a microplate reader. HUH02-derived preparations were added at bacterial cell equivalent concentrations of 1 × 10^6^–1 × 10^8^ CFU/mL, corresponding to approximate bacteria-to-cell ratios of 33:1, 333:1, and 3333:1.

### 2.6. Reverse Transcription Quantitative Real-Time PCR (RT-qPCR)

Total RNA was extracted from cells using a Total RNA Extraction Mini Kit (APbio, Namyangju, Republic of Korea) according to the manufacturer’s instructions. Genomic DNA contamination was removed by on-column DNase treatment using DNase I (Qiagen, Hilden, Germany). First-strand cDNA was synthesized from the extracted RNA using an APamp™ RT-PCR Premix 2X Kit (Applied Biosystems, Foster City, CA, USA). Reverse transcription quantitative real-time PCR (RT-qPCR) was performed using SYBR Green PCR Mix (APbio) and the QuantStudio™ 1 Real-Time PCR System (Applied Biosystems, Foster City, CA, USA). The amplification conditions and data analysis were performed as previously described. Relative gene expression levels were normalized to GAPDH expression and are presented as log2-transformed values. Primer sequences were designed using Primer3web (version 4.1.0) software (https://primer3.ut.ee/) and are listed in Table 1.

### 2.7. Western Blot Analysis

Total protein was extracted from RAW 264.7 and B16F1 cells using RIPA buffer (Thermo Fisher Scientific, Philadelphia, PA, USA) supplemented with a protease inhibitor cocktail (Roche, Mannheim, Germany). Protein concentrations were measured, and equal amounts of protein (30 μg per sample) were separated by 10% SDS-polyacrylamide gel electrophoresis using polyacrylamide gels (Bio-Rad, Hercules, CA, USA; #456–1096). The separated proteins were transferred to polyvinylidene fluoride (PVDF) membranes. After blocking, the membranes were incubated with primary antibodies overnight (at least 14 h) at 4 °C. Following washing, the membranes were incubated with the appropriate secondary antibodies for 1 h at room temperature (24 °C). Protein bands were visualized using Western Pico chemiluminescent substrate (Thermo Scientific, Rockford, IL, USA), and images were acquired using a Bright™ imaging system (Thermo Fisher Scientific, Waltham, MA, USA). β-actin was used as the loading control. Detailed information on the primary antibodies and their dilution ratios is provided in Table 2.

### 2.8. Wound Healing Assay

A wound healing assay was performed to evaluate cell migration. HaCaT cells were seeded into 24-well plates at a density of 5 × 10^4^ cells/well and cultured for 24 h until a confluent monolayer was formed. A linear scratch was created in the cell monolayer using a sterile Scar™ Scratcher (SPL Life Science, Pocheon, Republic of Korea). Detached cells were removed by washing the wells three times with Dulbecco’s Phosphate-Buffered Saline (DPBS; Welgene, Gyeongsan, Republic of Korea). Fresh culture medium containing HUH02-HK, HUH02-2psi, or HUH02-4psi at concentrations of 1 × 10^6^, 1 × 10^7^, and 1 × 10^8^ CFU/cm^2^, respectively, was added, and the cells were incubated for an additional 24 h. Images of the scratch area were captured using an inverted microscope (Nikon E-800; Nikon, Tokyo, Japan). Wound closure was quantified using ImageJ software (version 1.54d) and expressed as a percentage of the initial wound area at time 0.

### 2.9. Transepithelial Electrical Resistance (TEER) Measurement

To evaluate the effect of HUH02 preparations on epithelial barrier integrity, transepithelial electrical resistance (TEER) was measured in HaCaT cells. HaCaT cells were seeded at a density of 1 × 10^5^ cells/insert into 12-well Transwell inserts containing polyethylene membranes with a pore size of 0.4 μm (SPL Life Science, Pocheon, Republic of Korea) and cultured for 24 h to allow cell attachment and monolayer formation. The cells were then treated with HUH02-HK, HUH02-2psi, or HUH02-4psi at concentrations of 1 × 10^6^, 1 × 10^7^, or 1 × 10^8^ CFU/cm^2^ and incubated for an additional 24 h. Before TEER measurement, the culture medium was replaced to eliminate any contribution of the HUH02 preparations to electrical resistance. The plates were then equilibrated at room temperature (24 °C) for 30 min to minimize temperature-dependent variation in resistance. TEER was measured using an EVOM3 epithelial voltohmmeter (World Precision Instruments, Sarasota, FL, USA) equipped with STX electrodes. Resistance was measured at three independent locations within each insert, and the average value was calculated. The resistance measured in cell-free inserts containing culture medium was subtracted from each sample. TEER values were normalized to the membrane surface area (1.13 cm^2^) using the following equation:

TEER (Ω⋅cm^2^) = (Rsample − Rblank) × 1.13 cm^2^

### 2.10. Melanin Content Assay

B16F1 cells were seeded into 6-well plates at a density of 4 × 10^4^ cells/well and cultured for 24 h under standard culture conditions. For all B16F1 cell experiments, trypsin-EDTA was diluted 1:1 with DPBS before use. After cell attachment, the culture medium was replaced with fresh medium containing α-melanocyte-stimulating hormone (α-MSH; Sigma-Aldrich, St. Louis, MO, USA) at 100 nM, and cells were pretreated for 1 h. Subsequently, the cells were treated with arbutin (Sigma-Aldrich; 1 mM), HUH02-HK, HUH02-2psi and 4psi (1 × 10^7^ CFU/mL) and incubated for 5 days. Following treatment, the culture medium was removed, and the cells were harvested using trypsin-EDTA. The collected cells were centrifuged at 800× *g* for 5 min, washed twice with DPBS, and resuspended in 200 μL of 1 N NaOH. The cell suspensions were incubated at 60 °C for 1 h with continuous shaking at 500 rpm to dissolve melanin. Subsequently, 100 μL of each lysate was transferred to a 96-well plate, and melanin content was quantified by measuring absorbance at 405 nm using a microplate reader.

### 2.11. Whole-Genome Sequencing and Analysis

Genomic DNA from strain HUH02 was extracted using the Quick-DNA HMW MagBead kit (Zymo Research Corporation, Irvine, CA, USA) following lysozyme treatment (Sigma-Aldrich, St. Louis, MO, USA). DNA concentration was determined using the QuantiFluor dsDNA System (Promega, Madison, WI, USA) with a Victor Nivo Multimode Microplate Reader (PerkinElmer, Waltham, MA, USA). Whole-genome sequencing was performed by Macrogen Inc. (Seoul, Republic of Korea) using the Sequel II system (Pacific Biosciences, Menlo Park, CA, USA) and the NovaSeq 6000 system (Illumina, San Diego, CA, USA). Raw sequencing data quality was assessed based on the Q20 and Q30 metrics. Low-quality Illumina reads were filtered before genome assembly. Flye (v2.4.2) was used to assemble the HiFi reads [19]. The assembled genome was subsequently polished using Illumina reads with Pilon [20]. Genome annotation was performed using Prokka [21] with default parameters. A circular genome map was generated using the Proksee web platform [22]. The whole-genome sequence data have been submitted to the NCBI GenBank database under the submission ID SUB16207515 (“HUH02_WGS”) and assigned accession number JBYPZP000000000.

### 2.12. Taxonomic Identification and Comparative Analysis

Taxonomic identification of strain HUH02 was initially performed using in silico 16S rRNA gene analysis with ContEst16S [23] to screen for potential sequence contamination and assign a preliminary taxonomic affiliation. Genome-level taxonomic assignment was subsequently performed using the Genome Taxonomy Database [24] framework, and average nucleotide identity (ANI) values were calculated against closely related strains. Comparative genomic analyses were conducted using the BV-BRC platform (www.bv-brc.org) with *L. gasseri* ATCC 33323 (GCF_000014425.1) and *L. paragasseri* JCM 5343 as reference strains. Shared orthologous gene families (PGfams) were identified, and HUH02-specific gene families were defined relative to JCM 5343.

### 2.13. Safety Assessment Procedure

Phenotypical safety assessment of strain HUH02 yielded consistently favorable results across MICs and cytotoxicity (Table S1). In genomic analysis, antibiotic resistance genes were investigated using three complementary tools: (i) PlasmidFinder (v2.1) to detect plasmid-mediated resistance determinants transmitted via conjugation [25]; (ii) PHAST to identify prophage regions and assess the presence of resistance genes within intact or incomplete prophage sequences [26]; and (iii) Mobile Element Finder (v1.0.3) to detect mobile genetic elements (MGEs) that may harbor resistance or virulence genes [27]. Virulence potential was predicted using PathogenFinder2 [28], a protein language model (PLM)-based deep learning tool that assigns a pathogenicity score ranging from 0 (nonpathogenic) to 1 (pathogenic). Additionally, VirulenceFinder 2.0 [29] was used to screen for known virulence genes associated with *Listeria*, *Staphylococcus aureus*, *Escherichia coli*, and *Enterococcus* spp. For phage-related virulence determinant analysis, coding sequences were predicted with Prodigal v2.6.3. Prophage regions were identified by BLASTP (BLAST+ v2.17.0) against phage-derived proteins of the NCBI RefSeq viral release (*n* = 575,306). Virulence determinants were screened by BLASTP against the VFDB setB. All tools were performed with the default parameters and thresholds.

### 2.14. Structure and Function Prediction of Hypothetical Proteins

Three-dimensional structures of hypothetical proteins were predicted using AlphaFold2 [30] as implemented in ColabFold [31]. Multiple sequence alignments (MSAs) were generated using the MMseqs2-based search pipeline [32] through the ColabFold MSA server. Three sequence databases were queried in parallel: (i) UniRef30 as the primary sequence database; (ii) the environmental metagenomics composite database comprising BFD, MGnify30, MetaEuk30, and SMAG (hereafter referred to as BFD/env); and (iii) PDB70 for structural template identification. MSA filtering was performed using the following parameters: a maximum pairwise sequence identity of 0.95, a query coverage score (QSC) threshold of 0.8, and a maximum difference (diff) of 3000 sequences to maintain MSA diversity. Structure prediction was performed using the AlphaFold2-ptm model set (five models) with three recycles per prediction. For each protein, five predicted models were ranked according to the predicted local distance difference test (pLDDT) and predicted aligned error (PAE), and the top-ranked model (rank_001) was selected for downstream analyses. Unrelaxed PDB coordinate files were used for Foldseek structural searches [33]. Prediction confidence was assessed using both the residue-level pLDDT score (0–100) and the predicted TM-score (pTM; global fold confidence; 0–1). To infer protein function from the predicted three-dimensional structures, each query structure was searched against two reference databases: (i) PDB100, comprising all experimentally determined structures deposited in the Protein Data Bank [34], and (ii) AlphaFoldDB/Swiss-Prot (afdb50), which contains AlphaFold2-predicted structures for proteins in the Swiss-Prot database [35]. All searches were performed using the ‘3Di + AA’ search mode, which combines structural alphabet-encoded residue contacts (3Di descriptors) with conventional amino acid sequence information to maximize sensitivity for detecting structural homologs regardless of sequence conservation. For each query, the hit with the highest probability score across both databases was designated as the best structural homolog.

### 2.15. Statistical Analysis

Statistical analyses were performed using IBM SPSS Statistics version 30.0 (IBM Corp., Armonk, NY, USA). All data are presented as the mean ± standard deviation (SD). Comparisons among multiple groups were performed using one-way analysis of variance (ANOVA), followed by Dunnett’s multiple comparisons test. For inflammatory and melanogenesis induction models, the LPS-treated and α-MSH-treated groups were considered the respective model control groups, and statistical comparisons were performed against these groups. Statistical significance was defined as * *p* < 0.05, ** *p* < 0.01, and *** *p* < 0.001 compared with the control group. For comparison with the negative control group (LPS or α-MSH), significance was denoted as # *p* < 0.05, ## *p* < 0.01, and ### *p* < 0.001. Graphs were generated using GraphPad Prism version 9.3 (GraphPad Software, San Diego, CA, USA).

## 3. Results

### 3.1. Morphological Characteristics of Breast Milk-Derived Live HUH02 Cells, Heat-Killed HUH02 Cells, and Microfluidized HUH02 Cells

**Figure 1:**
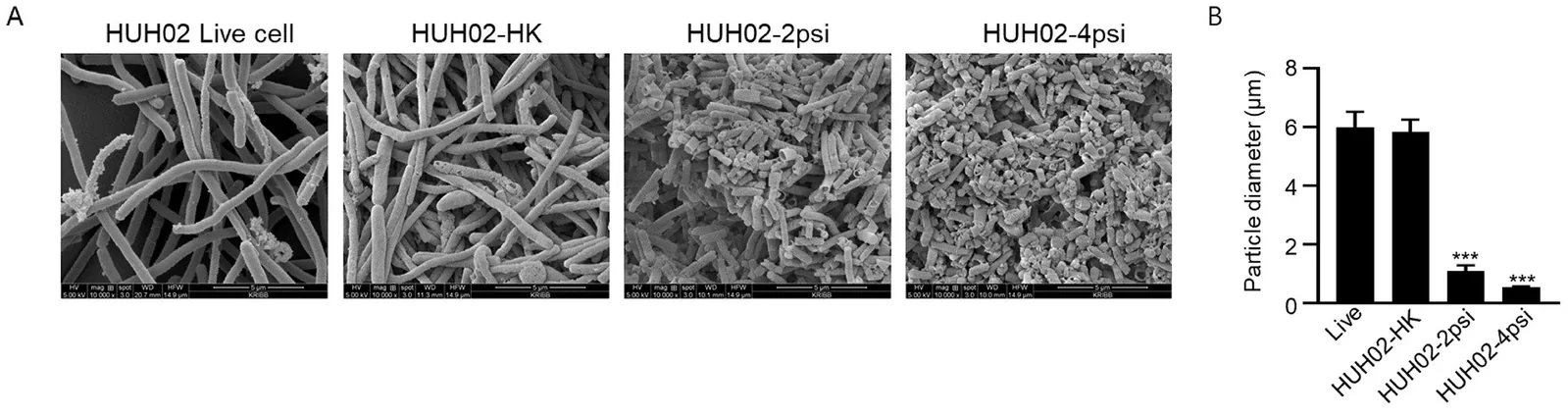
Morphological characterization of HUH02-derived preparations. (**A**) FE-SEM images of live HUH02 cells, heat-killed HUH02 cells (HUH02-HK), and microfluidized heat-killed HUH02 preparations processed at 20,000 psi (HUH02-2psi) or 40,000 psi (HUH02-4psi). Scale bar = 5 μm. (**B**) Comparison of cell lengths of HUH02 preparations (*n* = 10). Data are presented as the mean ± standard deviation (SD). Statistical significance is indicated as *** *p* < 0.001.

The morphological characteristics of live HUH02 cells, heat-killed HUH02 cells (HUH02-HK), and microfluidized HUH02 preparations (HUH02-2psi and HUH02-4psi) were investigated using FE-SEM. Representative images are shown in Figure 1.

Compared with live HUH02 cells (Figure 1A), HUH02-HK cells exhibited partial disruption of the cell envelope, indicating morphological alterations induced by heat treatment. In contrast, the microfluidized HUH02 preparations (HUH02-2psi and HUH02-4psi) exhibited more pronounced structural alterations, including surface damage and reduced cell length, following high-pressure homogenization. The average cell length of live HUH02 cells was 5.99 ± 0.54 μm, whereas HUH02-HK cells showed a comparable cell length of 5.82 ± 0.43 μm, with no significant difference between the two groups. In contrast, the average cell lengths of HUH02-2psi and HUH02-4psi were markedly reduced to 1.096 ± 0.202 μm and 0.549 ± 0.036 μm, respectively, compared with those of live HUH02 and HUH02-HK cells (Figure 1B).

### 3.2. Effects of HUH02-Derived Preparations on Cell Viability in RAW264.7 Macrophages, HaCaT Keratinocytes, and B16F1 Melanoma Cells

**Figure 2:**
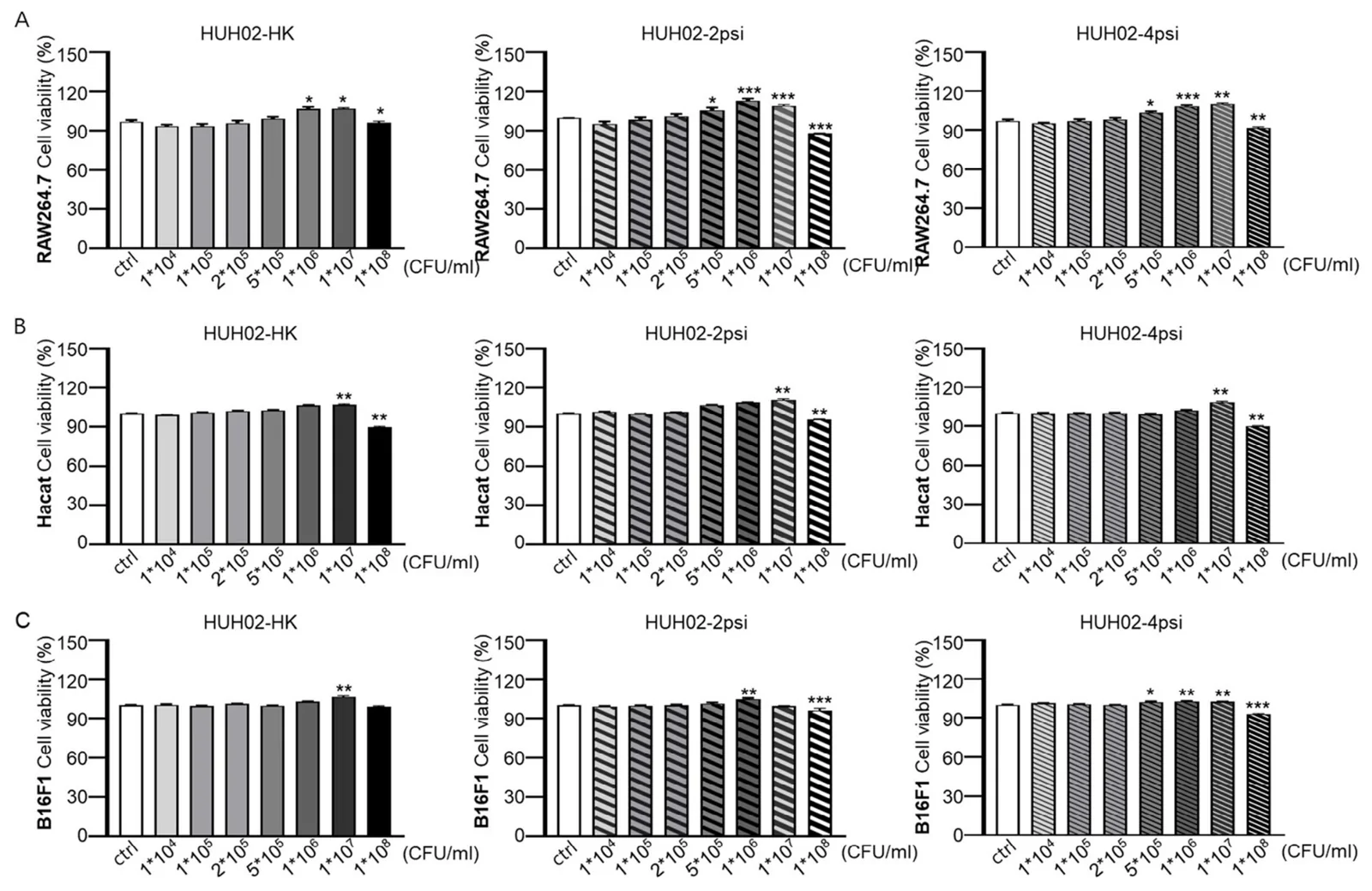
Cytotoxicity of HUH02-HK, HUH02-2psi, and HUH02-4psi in RAW264.7, HaCaT, and B16F1 cells. Cell viability was assessed in (**A**) RAW264.7 macrophages, (**B**) HaCaT keratinocytes, and (**C**) B16F1 melanoma cells following treatment with HUH02-HK, HUH02-2psi, or HUH02-4psi at concentrations ranging from 10^4^ to 10^8^ CFU/mL (*n* = 5). Data are presented as the mean ± standard deviation (SD). Statistical significance is indicated as * *p* < 0.05, ** *p* < 0.01, and *** *p* < 0.001.

The cytotoxicity of HUH02-derived preparations, including a heat-killed HUH02 (HUH02-HK) and microfluidized HUH02 preparations (HUH02-2psi and HUH02-4psi), was evaluated in RAW264.7 macrophages, HaCaT keratinocytes, and B16F1 melanoma cells. Cell viability was assessed following treatment with each preparation at concentrations ranging from 1 × 10^6^ to 1 × 10^8^ CFU/mL (Figure 2). In RAW264.7 macrophages, HUH02-HK, HUH02-2psi, and HUH02-4psi significantly increased cell viability compared with the untreated control at concentrations of 1 × 10^6^ CFU/mL or higher. At 1 × 10^7^ CFU/mL, all HUH02-derived preparations increased cell viability by approximately 6–10%. However, at the highest concentration (1 × 10^8^ CFU/mL), all preparations significantly reduced cell viability by approximately 4–12% compared with the control group (Figure 2A). Similarly, in HaCaT keratinocytes, treatment with HUH02-HK, HUH02-2psi, and HUH02-4psi increased cell viability. At 1 × 10^7^ CFU/mL, all preparations improved cell viability by approximately 6–10% compared with the control group. Conversely, at a concentration of 1 × 10^8^ CFU/mL, cell viability significantly decreased by approximately 5–11% (Figure 2B). In B16F1 melanoma cells, treatment with HUH02-HK, HUH02-2psi, or HUH02-4psi at 1 × 10^7^ CFU/mL did not significantly affect cell viability. However, at 1 × 10^8^ CFU/mL, HUH02-2psi and HUH02-4psi significantly reduced cell viability by approximately 4–8% compared with the control group (Figure 2C). These results indicated that HUH02-derived preparations exhibited minimal cytotoxicity in RAW264.7, HaCaT, and B16F1 cells at concentrations up to 1 × 10^7^ CFU/mL. Therefore, 1 × 10^7^ CFU/mL was selected for subsequent functional analyses.

### 3.3. Anti-Inflammatory Effects of HUH02-Derived Preparations in LPS-Stimulated RAW264.7 Macrophages

**Figure 3:**
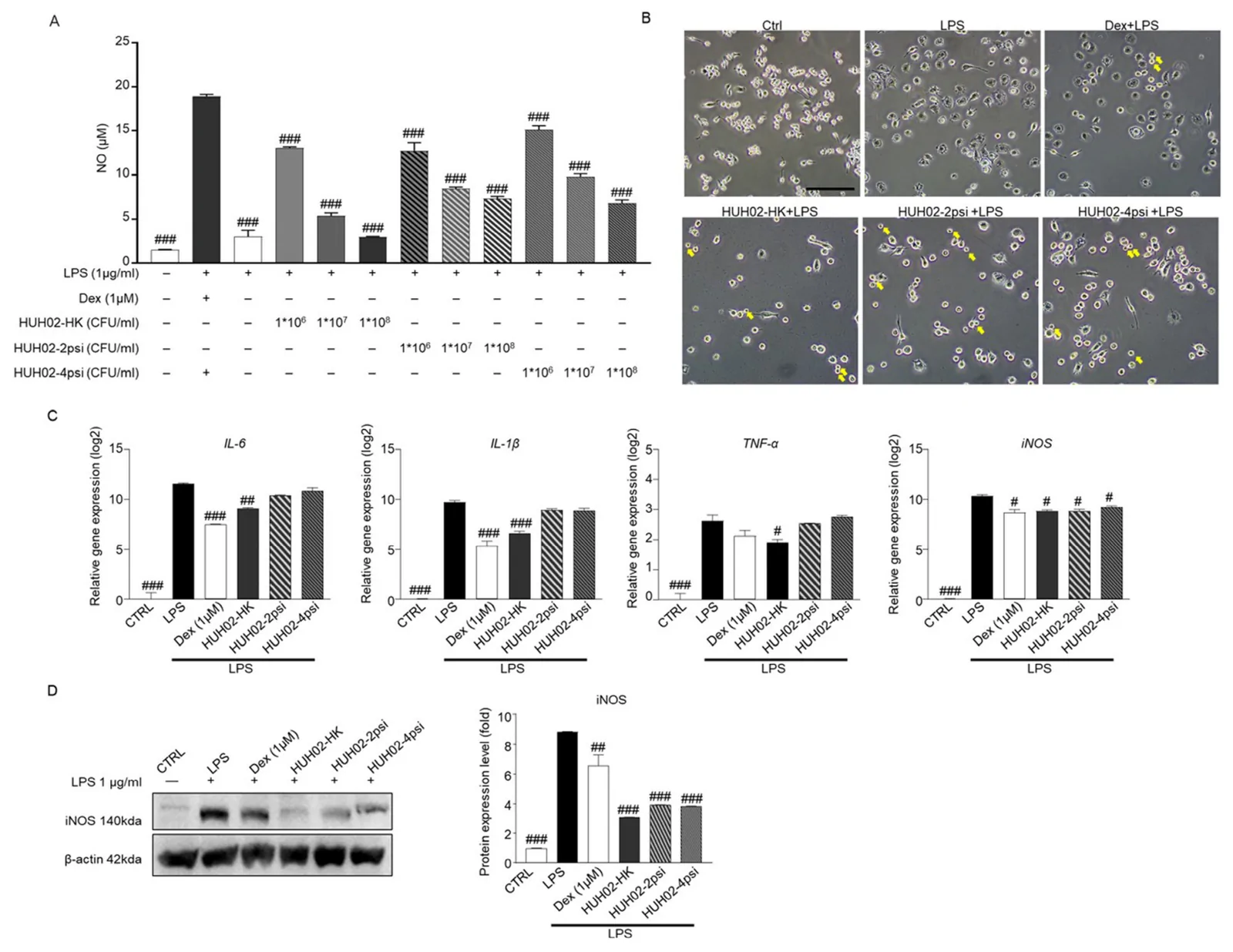
Anti-inflammatory effects of HUH02-HK, HUH02-2psi, and HUH02-4psi in LPS-stimulated RAW264.7 macrophages. (**A**) Nitric oxide (NO) production following treatment with LPS and HUH02 preparations (*n* = 5). (**B**) Morphological changes in RAW264.7 cells following treatment with LPS and HUH02 preparations. Scale bar = 100 μm. (**C**) Relative mRNA expression levels of inflammation-related genes, including *IL-6*, *IL-1β*, *TNF-α*, and *iNOS*, following treatment with LPS and HUH02 preparations (1 × 10^7^ CFU/mL) (*n* = 5). (**D**) iNOS protein expression levels in RAW264.7 cells under the same treatment conditions (*n* = 3). Data are presented as mean ± standard deviation (SD). Significant differences compared with the LPS-treated group (negative control) are indicated as # *p* < 0.05, ## *p* < 0.01, and ### *p* < 0.001.

The anti-inflammatory activities of HUH02-derived preparations were evaluated in LPS-stimulated RAW264.7 macrophages (Figure 3). Nitric oxide (NO) production was measured to determine whether HUH02-HK, HUH02-2psi, and HUH02-4psi attenuated LPS-induced inflammatory responses. LPS stimulation markedly increased NO production to more than 18 μM compared with the untreated control group. In contrast, dexamethasone (DEX, 1 μM), used as a positive control, significantly reduced NO production to below 3 μM. Compared with the LPS-treated group, HUH02-HK significantly decreased NO production at concentrations of 1 × 10^6^, 1 × 10^7^, and 1 × 10^8^ CFU/mL, with NO levels of 13.04 ± 0.15, 5.40 ± 0.30, and 2.94 ± 0.05 μM, respectively. Similarly, HUH02-2psi significantly reduced NO production to 12.74 ± 0.94, 8.44 ± 0.20, and 7.30 ± 0.25 μM at the corresponding concentrations, whereas HUH02-4psi reduced NO levels to 15.12 ± 0.45, 9.82 ± 0.30, and 6.80 ± 0.40 μM (Figure 3A). Based on the cell viability results, subsequent anti-inflammatory experiments were performed using 1 × 10^7^ CFU/mL, which did not exhibit cytotoxic effects. Morphological changes in RAW264.7 macrophages were examined following treatment with LPS alone or in combination with HUH02-derived preparations (Figure 3B). LPS stimulation induced characteristic macrophage activation, including enlarged and irregular cell morphology, increased cell spreading, and prominent pseudopodia-like and dendritic extensions compared with untreated cells. In contrast, co-treatment with DEX, HUH02-HK, HUH02-2psi, or HUH02-4psi maintained a morphology similar to that of the untreated control group, characterized by smaller, rounded cells with smooth surfaces (yellow arrows). To further investigate the anti-inflammatory effects at the molecular level, the expression of inflammation-related genes, including *IL-6*, *IL-1β*, *TNF-α*, and *iNOS*, was analyzed. Treatment with HUH02-HK at 1 × 10^7^ CFU/mL significantly reduced the mRNA expression of IL-6, IL-1β, and TNF-α compared with the LPS-treated group. In addition, all HUH02-derived preparations significantly decreased iNOS mRNA expression compared with the LPS-treated group (Figure 3C). Consistent with the gene expression results, iNOS protein expression was significantly reduced following treatment with HUH02-HK, HUH02-2psi, and HUH02-4psi at 1 × 10^7^ CFU/mL compared with the LPS-treated group. These findings suggested that HUH02-derived preparations effectively suppressed LPS-induced inflammatory responses in RAW264.7 macrophages.

### 3.4. Enhancement of Epithelial Barrier Integrity by HUH02-Derived Preparations in HaCaT Keratinocytes

**Figure 4:**
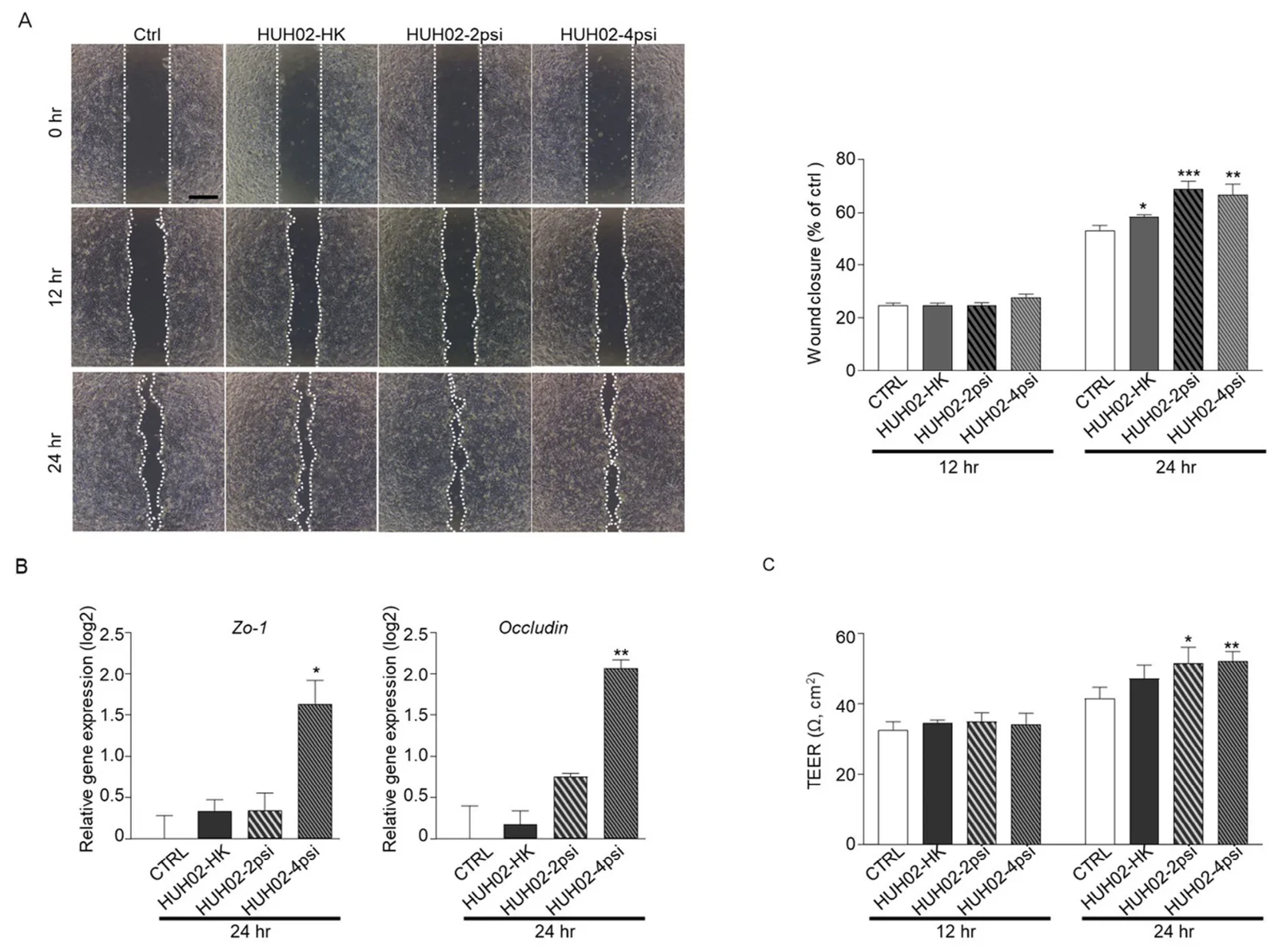
Effects of HUH02-HK, HUH02-2psi, and HUH02-4psi on wound healing and epithelial barrier integrity in HaCaT keratinocytes. (**A**) Representative images and quantitative analysis of wound closure in HaCaT cells treated with HUH02 preparations (1 × 10^7^ CFU/mL) for 12 and 24 h after scratch induction (*n* = 5). Scale bar = 500 μm. (**B**) Relative mRNA expression levels of the tight junction-related genes *ZO-1* and *Occludin* following treatment with HUH02 preparations (*n* = 5). (**C**) Transepithelial electrical resistance (TEER) values in HaCaT cells treated with HUH02 preparations for 12 and 24 h. Data are presented as mean ± standard deviation (SD). Statistical significance is indicated as * *p* < 0.05, ** *p* < 0.01, and *** *p* < 0.001. (*n* = 5).

The effects of HUH02-derived preparations on epithelial wound healing and barrier integrity were evaluated in HaCaT keratinocytes using wound closure and transepithelial electrical resistance (TEER) assays (Figure 4). HaCaT cells were treated with HUH02-HK, HUH02-2psi, and HUH02-4psi at 1 × 10^7^ CFU/mL, and wound closure and epithelial barrier-related markers were analyzed. In the wound closure assay, no significant differences were observed among the treatment groups during the initial 0–12 h after scratch induction. However, after 24 h, HUH02-derived preparations significantly enhanced wound closure compared with the untreated control group. The wound closure rates were 53.00 ± 1.84% in the control group, 58.48 ± 0.84% in the HUH02-HK group, 68.98 ± 2.84% in the HUH02-2psi group, and 66.92 ± 3.92% in the HUH02-4psi group (Figure 4A). Among the tested preparations, the microfluidized derivatives (HUH02-2psi and HUH02-4psi) promoted greater wound closure than HUH02-HK. To investigate the effects of HUH02-derived preparations on epithelial barrier-associated factors, the mRNA expression of the tight junction-related genes *ZO-1* and *Occludin* was analyzed. Treatment with HUH02-HK, HUH02-2psi, and HUH02-4psi at 1 × 10^7^ CFU/mL significantly increased the expression levels of both genes compared with the untreated control group (Figure 4B). The effects of HUH02-derived preparations on epithelial barrier function were further evaluated by measuring TEER in HaCaT cell monolayers. No significant differences were observed among the treatment groups during the initial 0–12 h. However, after 24 h, TEER increased from 41.60 ± 3.14 Ω·cm^2^ in the control group to 47.20 ± 3.93 Ω·cm^2^, 51.60 ± 4.45 Ω·cm^2^, and 52.23 ± 2.62 Ω·cm^2^ in the HUH02-HK, HUH02-2psi, and HUH02-4psi groups, respectively. Notably, HUH02-2psi and HUH02-4psi significantly increased TEER compared with the control group (Figure 4C). These findings indicated that HUH02-derived preparations, particularly the microfluidized derivatives, promoted epithelial wound recovery and enhanced barrier integrity by increasing tight junction-associated gene expression and improving functional epithelial resistance.

### 3.5. Inhibitory Effects of HUH02-Derived Preparations on Melanogenesis in B16F1 Melanoma Cells

**Figure 5:**
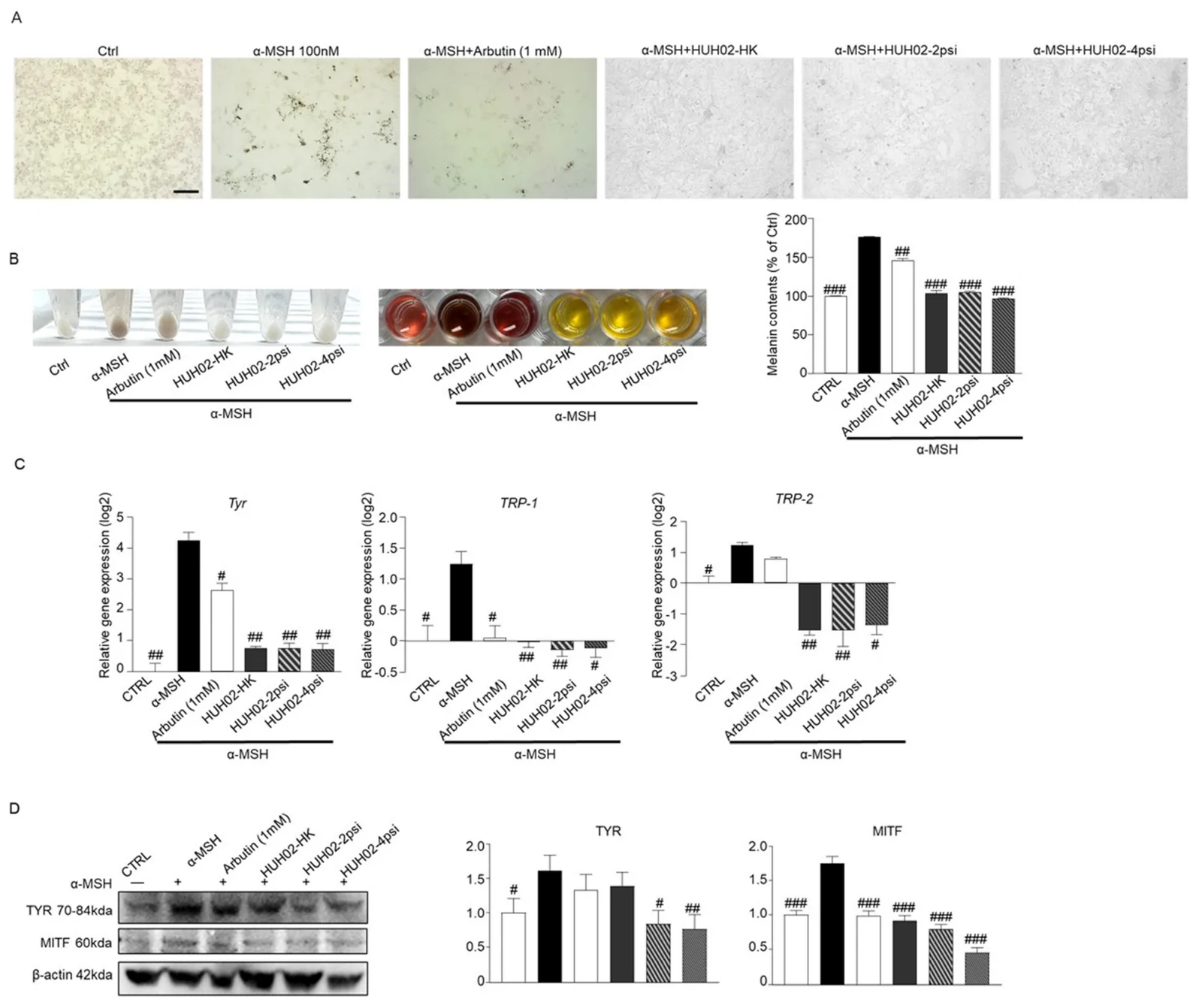
Melanogenesis-inhibitory effects of HUH02-HK, HUH02-2psi, and HUH02-4psi in α-MSH-stimulated B16F1 melanoma cells. (**A**) Representative images showing morphological changes associated with melanogenesis following treatment with α-MSH and HUH02 preparations (1 × 10^7^ CFU/mL) for 5 days (*n* = 5). scale bar = 200 μm. (**B**) Representative images of cell pellets, culture medium pigmentation, and melanin production following treatment (*n* = 5). (**C**) Relative mRNA expression levels of melanogenesis-related genes, including *Tyr*, *TRP-1*, and *TRP-2*, after 5 days of treatment (*n* = 5). (**D**) Protein expression levels of melanogenesis-related proteins TYR and MITF after 5 days of treatment (*n* = 3). Data are presented as mean ± standard deviation (SD). Significant differences compared with the α-MSH-only treated group (negative control) are indicated as # *p* < 0.05, ## *p* < 0.01, and ### *p* < 0.001.

The effects of HUH02-derived preparations on melanogenesis were evaluated in B16F1 melanoma cells (Figure 5). B16F1 cells were treated with HUH02-HK, HUH02-2psi, and HUH02-4psi at 1 × 10 CFU/mL for 5 days, and changes in melanin production were assessed. Following treatment, cell morphology and melanin accumulation were examined using bright-field microscopy (Figure 5A). Compared with the α-MSH-stimulated group (100 nM), which served as the positive control for melanogenesis induction, HUH02-derived preparations visibly reduced melanin accumulation in B16F1 cells. The cells were subsequently collected by centrifugation, and intracellular melanin content was quantified (Figure 5B). Relative melanin content increased significantly in the α-MSH-treated group (176.00 ± 0.49%) compared with the untreated control group. In contrast, treatment with HUH02-HK, HUH02-2psi, and HUH02-4psi reduced melanin accumulation to 103.45 ± 3.94%, 105.42 ± 0.99%, and 96.55 ± 0.98%, respectively. All HUH02-derived preparations significantly reduced melanin content compared with the α-MSH-stimulated group (Figure 5B). To further investigate the molecular mechanisms underlying these antimelanogenic effects, the mRNA expression of the melanogenesis-associated genes *Tyr*, *TRP-1*, and *TRP-2* was analyzed. Treatment with HUH02-HK, HUH02-2psi, and HUH02-4psi at 1 × 10^7^ CFU/mL significantly reduced the expression of all three genes compared with the α-MSH-stimulated group (Figure 5C). Consistent with the gene expression results, protein expression analysis revealed that HUH02-2psi and HUH02-4psi significantly decreased the protein levels of TYR and MITF compared with the α-MSH-treated group (Figure 5D). These results suggested that HUH02-derived preparations, particularly the microfluidized derivatives, suppressed melanogenesis by reducing melanin accumulation and downregulating key melanogenic regulators in B16F1 melanoma cells.

### 3.6. General Genome Characteristics and Annotation

**Figure 6:**
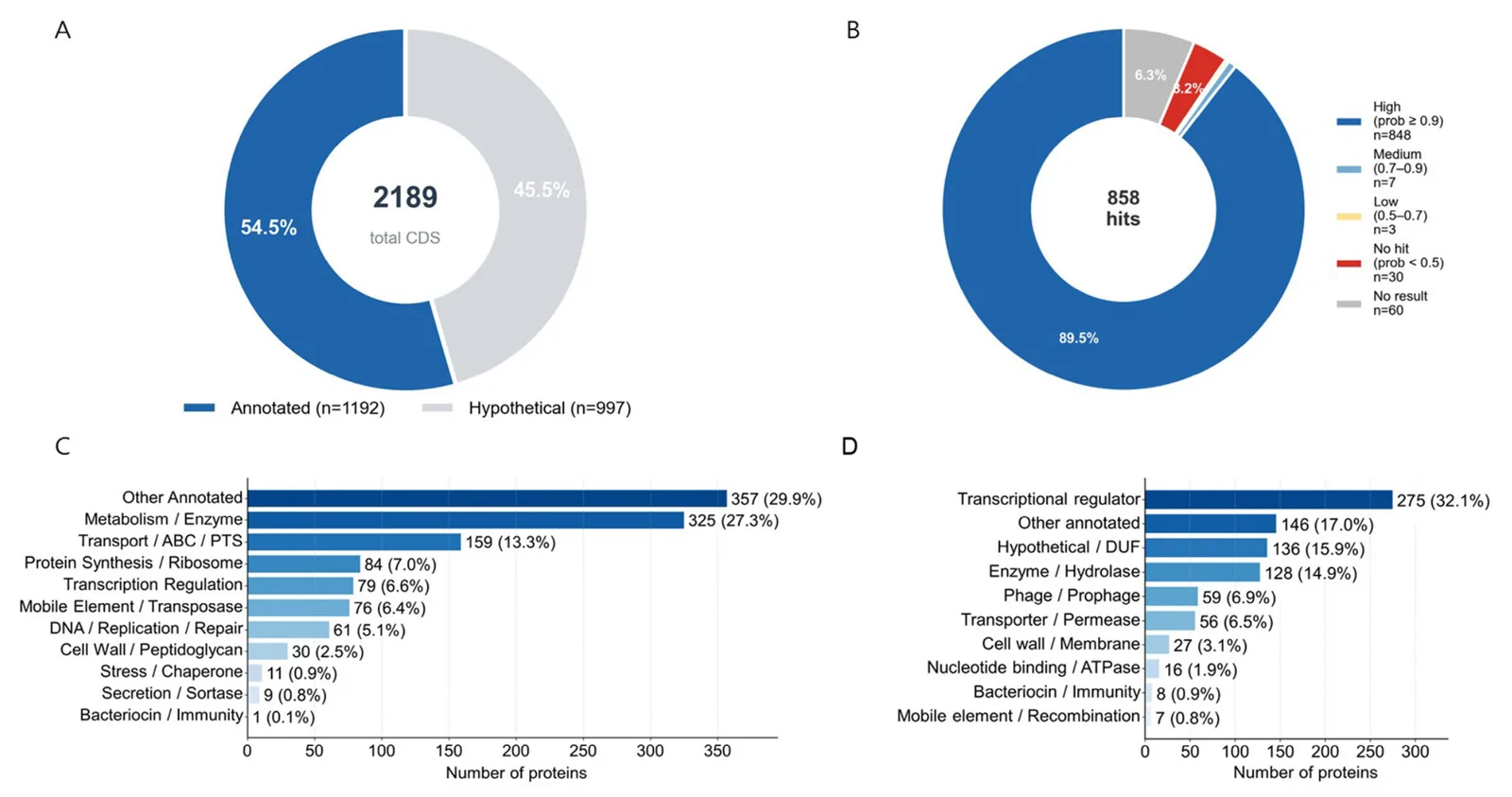
Functional analysis overview of the *L. paragasseri HUH02* genome. (**A**) Distribution of protein-coding sequences (CDSs). (**B**) Distribution of predicted structural homology for hypothetical proteins analyzed using Alphafold and Foldseek. The percentage of High is 89.5%, Medium is 1%, No results is 6.3%, and No hit is 3.2%. (**C**) Functional category distribution of annotated proteins (*n* = 1192). (**D**) Functional category distribution of hypothetical proteins with significant structural homology hits (*n* = 858).

The genome of *L. paragasseri* HUH02 was assembled into three contigs with a total length of approximately 2.17 Mb. The largest contig was 1,862,766 bp and served as the N50 value, indicating a highly contiguous assembly. The assembled genome had a GC content of 34.9%, consistent with the reported genomic composition of *L. paragasseri*. No plasmid sequences were identified. After quality filtering, the Illumina short reads achieved Q20 and Q30 scores of 98.92% and 95.38%, respectively, indicating high base-calling accuracy. Structural RNA genes were annotated, yielding 15 rRNA genes arranged in five complete 16S–23S–5S operons, 72 tRNA genes, one tmRNA, and two ncRNA genes. Genome annotation identified 2189 coding sequences distributed across three contigs. Of these, 1192 (54.5%) received functional annotation, whereas 997 (45.5%) remained classified as hypothetical proteins (Figure 6A). Among the hypothetical proteins, 948 with sequence length ≥ 30 amino acids were selected for downstream structural prediction and functional analysis (Figure 6B). The functional categories and counts of the annotated CDSs and functionally predicted hypothetical proteins are shown in Figure 6C,D.

### 3.7. Taxonomic Identification

ContEst16S analysis identified five 16S rRNA gene fragments in the HUH02 genome, all of which clustered with *L. paragasseri* in the phylogenetic tree, with no evidence of sequence contamination. GTDB-based taxonomy assignment further confirmed the species identity. HUH02 shared 98.5% ANI and 87.34% alignment coverage with *L. paragasseri* GCF_003584685.1, exceeding the 95% ANI threshold for species demarcation. In contrast, the closely related *L. gasseri* ATCC 33323 showed only 93.6% ANI and < 80% alignment coverage, confirming clear species-level differentiation. Whole-genome phylogenetic analysis placed HUH02 within a well-supported clade containing *L. paragasseri* reference strains, distinct from *L. gasseri*, *L. johnsonii*, and other members of the *L. acidophilus* group. Collectively, these results conclusively identified strain HUH02 as *L. paragasseri* (Figure S3).

### 3.8. Safety Assessment

PlasmidFinder analysis detected no plasmid replicon sequences in any of the screened databases, indicating the absence of conjugative plasmid-mediated antibiotic resistance. PHAST analysis identified seven prophage regions in the HUH02 genome, including two intact regions (score > 90) and five incomplete or questionable regions. The 108 proteins encoded within the intact prophage regions were exclusively associated with phage structural and biological functions, and no antibiotic resistance genes were identified in any prophage region. Mobile Element Finder detected 111 MGEs distributed across contig1 (*n* = 107) and contig2 (*n* = 4). None of the identified MGEs was associated with antibiotic resistance or virulence determinants. PathogenFinder2 predicted a mean pathogenicity score of 0.2268 (std = 0.1135) across four independent neural network modules. This score was well below the pathogenicity threshold and classified HUH02 as a human nonpathogen. VirulenceFinder identified no virulence genes in the *Listeria*, *S. aureus*, *E. coli*, and *Enterococcus* databases, confirming the absence of known virulence factors. Five prophage regions totaling 153.4 kb (7.1% of the genome) were detected in strain HUH 02. Two were intact (64.2 and 26.7 kb), each carrying a complete morphogenesis module (terminase, portal, capsid, and tail) and a holin–endolysin lysis cassette. Their closest relatives were the temperate *Lactobacillus* siphovirus. No phage-related virulence determinants were identified. None of the prophage genes was annotated as a toxin, hemolysin, cytolysin, enterotoxin or superantigen. The 35 VFDB-positive proteins all corresponded to conserved housekeeping or exopolysaccharide-biosynthesis functions and to bile salt hydrolase, with no hemolysin or cytolysin structural genes, enterotoxin, or superantigen detected. Taken together, these results indicated that no genomic markers associated with safety concerns were detected in *L. paragasseri* HUH02.

### 3.9. Comparative Genomic Analysis

Comparative genomic analysis of HUH02 with *L. gasseri* ATCC 33323 and *L. paragasseri* JCM 5343 identified genomic regions that differentiated HUH02 from both reference strains (Figure S1). Relative to *L. paragasseri* JCM 5343, HUH02 harbored 35 unique protein families (PGfam). Notable among these were genes encoding a complete CRISPR-Cas9 system (Cas9, Cas1, and Cas2), phage-associated elements (antirepressor, integrase, major capsid protein, tail length tape-measure protein, and replication initiation proteins), and metabolic enzymes, including alpha-L-rhamnosidase and L-rhamnose permease RhaY (involved in rhamnose utilization), maltodextrin glucosidase (involved in maltodextrin metabolism), and 2,3-butanediol dehydrogenase (involved in acetoin-butanediol conversion). Class Ib ribonucleotide reductase genes (both the alpha and beta subunits) were also uniquely present in HUH02, suggesting aerobic adaptation.

### 3.10. Analysis of Inflammation-Related Proteins

Across the annotated proteins and functionally predicted hypothetical proteins, 231 proteins were identified as putatively inflammation-relevant, including 131 with pro-inflammatory potential, 35 with anti-inflammatory potential, and 65 classified as dual or context-dependent (Table 3). Overall, the genomic repertoire of *L. paragasseri* HUH02 suggests a conditionally proinflammatory profile alongside substantial anti-inflammatory potential. The predicted pro-inflammatory potential was primarily associated with an extensive complement of integrated prophage-related proteins (*n* = 57, identified among the hypothetical proteins) and cell wall biosynthesis-related proteins (*n* = 36, identified by Prokka annotation). However, HUH02 has anti-inflammatory mechanisms (*n* = 35), including exopolysaccharides (EPS) biosynthesis gene clusters that dampen TLR-mediated signaling; multiple bacteriocin operons, including lactacin-F and helveticin-J, which can reshape the local microbiome; and a robust thioredoxin-based redox buffering system. The coexistence of opposing elements is not unusual among *Lactobacillus* strains and does not inherently contraindicate probiotic use. Rather, these findings highlighted the importance of experimental validation under physiologically relevant conditions.

## 4. Discussion

Breast milk harbors a diverse microbial community that contributes to neonatal gut microbiota establishment and immune maturation, making it an important source of beneficial microorganisms [36]. Lactobacillus species are among the representative members of this microbiota and are known to promote immune regulation, epithelial homeostasis, and the production of bioactive metabolites [37]. Breast milk-derived *Lactobacillus* strains have been reported to exhibit antimicrobial activity, inhibit pathogen colonization, support epithelial barrier function, modulate the gut microbiota, synthesize vitamins, and enhance immune function [38]. In the present study, whole-genome analysis identified strain HUH02 as *L. paragasseri*, a species previously classified within the *Lactobacillus gasseri* group but now recognized as a distinct taxon based on genomic divergence [39]. This genomic identification supports the evaluation of HUH02 as a strain with potential probiotic and postbiotic applications.

Postbiotics derived from inactivated microorganisms have gained attention because of their stability and safety advantages, including the elimination of risks associated with viability, horizontal gene transfer, and storage instability [40]. Heat-killed lactobacilli retain microbe-associated molecular patterns (MAMPs), such as peptidoglycan, lipoteichoic acid, and EPS, which interact with host pattern recognition receptors to regulate immune responses [41]. Several studies have reported that non-viable *Lactobacillus* retains immunomodulatory activity comparable to that of live cells, indicating that bacterial viability is not essential for these biological functions [42,43]. Consistent with these findings, HUH02-derived preparations retained biological activity after heat treatment and microfluidization. HUH02-HK, HUH02-2psi, and HUH02-4psi exhibited anti-inflammatory, epithelial barrier-supportive, and anti-melanogenic activities in RAW264.7 macrophages, HaCaT keratinocytes, and B16F1 cells, respectively. These findings suggest that the biological activities of the preparations are associated with bacterial structural components and/or intracellular molecules rather than bacterial proliferation. Genomic analysis further supported this possibility by identifying genes associated with EPS biosynthesis, bacteriocin production, and redox regulation. EPS, in particular, has been reported to contribute to immunomodulation and epithelial protection through regulation of Toll-like receptor signaling and barrier maintenance [41].

The cytotoxicity assessment demonstrated that the biological responses to HUH02-derived preparations differed among cell types. In RAW264.7 macrophages and HaCaT keratinocytes, treatment at concentrations up to 1 × 10^7^ CFU/mL not only showed minimal cytotoxicity but also resulted in a modest increase in cell viability. This response may reflect enhanced cellular metabolic activity or proliferation stimulated by postbiotic-derived bioactive molecules, including peptidoglycan, lipoteichoic acid, exopolysaccharides, and intracellular metabolites, which have been reported to regulate immune and epithelial cell functions [11,41,42]. In contrast, viability decreased at higher concentrations, suggesting that excessive exposure to bacterial components may induce cellular stress or exceed the concentration range compatible with cellular homeostasis [44]. B16F1 melanoma cells did not exhibit a comparable increase in viability, further indicating that cellular responses to HUH02-derived preparations depend on cell type. These differences may be related to differences in cellular physiology, receptor expression, and intracellular signaling pathways among macrophages, keratinocytes, and melanoma cells [45]. Collectively, these findings support the use of 1 × 10^7^ CFU/mL as an appropriate concentration for subsequent functional analyses while emphasizing the importance of considering cell-specific responses when evaluating postbiotic preparations.

FE-SEM analysis demonstrated that the extent of bacterial structural disruption differed according to the processing method. Heat treatment caused partial membrane damage, whereas microfluidization induced more extensive fragmentation and a marked reduction in bacterial cell length. Structural disruption is increasingly recognized as an important determinant of postbiotic activity because it can alter the accessibility and composition of bioactive bacterial components [46]. Heat-killed bacteria retain cell envelope components, such as peptidoglycan and lipoteichoic acid, which activate immune signaling via TLR2 [47]. These retained surface-associated components may contribute to the immunomodulatory activity observed with HUH02-HK. In contrast, microfluidization at 20,000 psi and 40,000 psi progressively disrupted the bacterial structure and may have increased the accessibility of intracellular peptides, metabolites, and nucleic acid-derived molecules [48]. Mechanical disruption of lactic acid bacteria has been shown to enhance the release of bioactive compounds and alter their biological activities [49]. Thus, the differences in biological activity observed among the HUH02 preparations may reflect not simply the extent of structural disruption but also differences in the types and relative availability of bioactive components generated or exposed by each processing method.

The anti-inflammatory activity of the HUH02-derived preparations was evaluated using LPS-stimulated RAW264.7 macrophages. LPS-induced macrophage activation is primarily mediated through TLR4 signaling and subsequent activation of NF-κB and MAPK pathways, leading to the production of inflammatory mediators such as NO, TNF-α, IL-6, IL-1β, and iNOS [50,51]. In the present study, all HUH02-derived preparations significantly reduced NO production and inflammatory cytokine expression while preserving normal macrophage morphology. Because iNOS expression is regulated by inflammatory signaling pathways, including NF-κB and MAPK, it is widely used as a marker of macrophage inflammatory activation [52]. The suppression of iNOS expression by all HUH02 preparations therefore supports their anti-inflammatory activity. Heat-killed Lactobacillus has been reported to modulate macrophage responses through TLR2-mediated recognition of bacterial surface components such as peptidoglycan and EPS [41]. Similarly, non-viable Lactobacillus species can suppress excessive cytokine production through immune receptor-mediated signaling [53]. Consistent with these findings, HUH02-HK exhibited the strongest anti-inflammatory activity, suggesting that preservation of bacterial surface-associated components may play a predominant role in regulating macrophage inflammatory responses. In contrast, microfluidized preparations may have increased the accessibility of intracellular components that contribute to other biological functions, including epithelial repair and pigmentation regulation. These findings indicate that the functional activity of HUH02-derived preparations is influenced by both the processing method and the bioactive components made accessible by that processing.

The skin serves as a physical and immunological barrier against microbial invasion and environmental stress [54]. Wound healing requires coordinated keratinocyte migration, proliferation, and formation of intercellular junctions [4]. Accordingly, enhanced wound closure and maintenance of epithelial barrier function are important indicators of skin-protective activity [55]. In the present study, HUH02-derived preparations accelerated wound closure in HaCaT keratinocytes, with stronger effects observed following microfluidization. The greater activity of the microfluidized preparations compared with HUH02-HK may be associated with increased accessibility of intracellular bioactive molecules released or exposed during mechanical disruption. Previous studies have demonstrated that processing can influence the composition and biological activity of postbiotics by promoting the release of proteins, peptides, and other functional metabolites [13,56]. These findings support the possibility that structural disruption of HUH02 increases the availability of components involved in epithelial repair.

HUH02-derived preparations also increased the expression of the tight-junction proteins ZO-1 and Occludin and increased TEER values, suggesting improved epithelial barrier function. Tight junctions are essential for regulating epithelial permeability, and their disruption is associated with inflammation and barrier dysfunction [57]. Increased expression of tight-junction proteins together with elevated TEER values provides evidence consistent with improved barrier function [58]. However, TEER measurements and gene expression analyses alone cannot fully characterize the structural and functional integrity of the epithelial barrier. Increased protein expression does not necessarily indicate appropriate localization or organization of tight-junction proteins at cell–cell junctions. Therefore, future studies should evaluate the cellular localization and organization of ZO-1 and Occludin using immunofluorescence analysis and should incorporate complementary paracellular permeability assays using appropriate fluorescent tracers. These approaches would provide more comprehensive structural and functional evidence for the barrier-protective effects of HUH02-derived preparations and further clarify their potential translational relevance.

The successful hybrid assembly of the HUH02 genome into only three contigs, representing a near-complete assembly, substantially improved downstream analyses. The safety assessment of HUH02 yielded consistently favorable results across all evaluated parameters. The absence of plasmid-borne, prophage-borne, or MGE-associated antibiotic resistance genes is an important criterion for the regulatory approval of probiotic strains and is consistent with the EFSA Qualified Presumption of Safety (QPS) criteria [59]. Although intact prophage regions were identified, the encoded proteins were exclusively associated with phage biology, and no antibiotic resistance or toxin-related genes were detected. The 111 MGEs identified represented the major contributors to genomic plasticity in HUH02; however, none carried antibiotic resistance or virulence genes. Elevated PLM scores have been previously reported in *Lactobacillus* strains and may reflect the presence of surface proteins or stress response elements that share structural similarity with virulence-associated proteins from distantly related organisms rather than indicating true virulence potential [28]. The functional gene repertoire of HUH02 highlighted multiple attributes relevant to probiotic applications. In particular, the presence of biosynthetic pathways for folate, riboflavin, thiamine, and vitamin B6 is noteworthy. The co-occurrence of mixed acid and lactate fermentation pathways, together with acetoin/butanediol metabolism, suggests metabolic flexibility that could support colonization under varying gut conditions. Comparative genomics revealed that HUH02 possessed several gene families absent from the type strain of *L. paragasseri* JCM 5343. The predominance of phage-associated gene differences between HUH02 and JCM 5343 is consistent with the known role of prophage elements in driving genomic diversification within *Lactobacillus* species [60]. A particularly noteworthy finding was that structure-based prediction identified 858 functional hits (90.5% hit rate; high confidence: 89.5%) among the 948 hypothetical proteins, revealing 106 inflammation-relevant proteins that would have been entirely invisible using conventional annotation-only approaches. These findings highlighted the added value of integrating AlphaFold2-based structural prediction with Foldseek homology searches to improve the functional annotation of bacterial genomes containing large numbers of hypothetical proteomes. Collectively, these genomic safety characteristics are consistent with internationally recognized safety assessment criteria, including the EFSA Qualified Presumption of Safety (QPS) framework, and support the continued evaluation of HUH02-derived preparations for cosmetic and healthcare applications.

A notable feature of the present study is the integration of genomic characterization with a comparative evaluation of HUH02-derived preparations generated using different processing methods. To the best of our knowledge, this is the first study to systematically compare the biological activities of heat-killed and microfluidized preparations of the specific breast milk-derived *L. paragasseri* HUH02 isolate across macrophage, keratinocyte, and melanoma cell models. The distinct biological profiles of HUH02-HK, HUH02-2psi, and HUH02-4psi indicate that processing conditions can influence the functional properties of the resulting preparations. HUH02-HK showed the strongest anti-inflammatory activity, whereas HUH02-2psi showed the greatest anti-melanogenic activity and HUH02-4psi most effectively promoted wound healing and epithelial barrier-related responses. These findings suggest that processing strategy may be considered when developing HUH02-derived postbiotic preparations for different skin-related applications. Nevertheless, the present study was conducted using in vitro models, and the specific bioactive components and molecular mechanisms responsible for the observed effects remain to be fully elucidated. Further studies using physiologically relevant models will be necessary to determine the efficacy, safety, and mechanistic basis of these effects.

## 5. Conclusions

HUH02 (*L. paragasseri*), isolated from breast milk, was confirmed using genome analysis and exhibited no detectable major safety-related genomic risks. Furthermore, HUH02-derived preparations retained their biological activity after both heat treatment and microfluidization. In RAW264.7 macrophages, HUH02 significantly reduced LPS-induced NO production and suppressed the expression of iNOS and pro-inflammatory cytokines. In HaCaT keratinocytes, HUH02 promoted wound closure and increased the expression of ZO-1 and Occludin, thereby improving epithelial barrier function. In B16F1 cells, HUH02 inhibited melanogenesis by reducing melanin production and downregulating MITF and other melanogenesis-related genes and proteins. Microfluidization induced greater structural disruption than heat treatment; however, the HUH02-2psi and HUH02-4psi treatments showed differential biological activities. Overall, these findings identify HUH02 as a promising breast milk-derived *L. paragasseri* strain with multifunctional postbiotic properties, including anti-inflammatory, skin barrier-supportive, wound-healing, and depigmenting potential. The distinct activities of the different HUH02 preparations further suggest that processing conditions may influence their functional properties and could be considered when developing postbiotic formulations for specific applications. Although the present findings were obtained using in vitro models, they provide a basis for further evaluation of the efficacy and safety of HUH02-derived preparations in more physiologically relevant models.

## Figures and Tables

**Table 1:** Primer sequences and PCR product sizes used for RT-qPCR analysis.

Gene	Forward Primer	Reverse Primer
*GAPDH (Mouse, 150 bp)*	5′-GTCGGTGTGAACGGATTTG-3′	5′-CTTGCCGTGGGTAGAGTCAT-3′
*IL-6 (Mouse, 174 bp)*	5′-TGATGCTGGTGACAACCACG-3′	5′-CAGAATTGCCATTGCACAACTC-3′
*IL-1β (Mouse, 116 bp)*	5′-ACCTTCCAGGATGAGGACATGA-3′	5′-CTAATGGGAACGTCACACACCA-3′
*TNF-α (Mouse, 89 bp)*	5′-CAGGCGGTGCCTATGTCTC-3′	5′-CGATCACCCCGAAGTTCAGTAG-3′
*iNOS (Mouse, 127 bp)*	5′-GTTCTCAGCCCAACAATACAAGA-3′	5′-GTGGACGGGTCGATGTCAC-3′
*Tyr (Mouse, 166 bp)*	5′-CCTGGAAACCATGACAAAGC-3′	5′-CTGCTATCCCTGTGAGTGGAC-3′
*TRP-1 (Mouse, 93 bp)*	5′-TGGTCTGTGAATCCTTGGAA-3′	5′-CATTTCCAGCTGGGTTTCTC-3′
*TRP-2 (Mouse, 132 bp)*	5′-CGTGCTGAACAAGGAATGC-3′	5′-CGAAGGATATAAGGGCCACTC-3′
*GAPDH (Human, 119 bp)*	5′-TCAAGAAGGTGGTGAAGCAG-3′	5′-AGCGTCAAAGGTGGAGGAG-3′
*Zo-1 (Human, 175 bp)*	5′-AGCCATTCCCGAAGGAGTTG-3′	5′-ATCACAGTGTGGTAAGCGCA-3′
*Occludin (Human, 365 bp)*	5′-CACATTTATGATGAGCAGCCCC-3′	5′-CCGCCAGTTGTGTAGTCTGT-3′

**Table 2:** List of antibodies for immunostaining and blotting.

Antibody	Manufacturer	Catalog Number	Dilution
β-actin	Santa Cruz Biotech	SC-47778	1:2000
iNOS	Cell signaling	20609	1:2000
TYR	Santa Cruz	SC-20035	1:2000
MITF	Santa Cruz	SC-515925	1:2000

**Table 3:** Inflammation-related proteins of *L. paragasseri* HUH02.

		Annotated ^a^		Hypothetical ^b^	
Role	Category	n	Key Proteins	n	Key Proteins
Pro-inflammatory	Phage, Prophage	5	Peptidyl-lysine N-acetyltransferase Ligase Glucosyl transferase Acetyltransferase-like- protein	57	Holin Lysin Hemolysin Phage anti-repressor Prophage proteins
	Toxin, Hemolysin	7	Glutamine- methyltransferase ATP-binding protein *LolD* Endoribonuclease toxin *MazF* Peptide chain release factors	14	Hemolysin III Salmolysin Toxin *RelE*, *HicA*, *YafQ*
	Virulence	2	Biofilm regulatory protein A Virulence factor B	5	Virulence protein E Adhesin Collagen-binding *BrkB*
	Cell Wall, TLR	35	Murein transglycosylase F Carboxypeptidase *DacA* Peptidoglycan Transglycosylase Transporters N-acetylmuramic acid/N-Acetylglucosamine kinase	4	LPXTG cell wall proteins Peptidoglycan-metalloendopeptidase
	Biogenic Amine	1	Ornithine decarboxylase	1	Carboxymuconolactone- decarboxylase
Anti-inflammatory	EPS, Capsule	5	Glycosyltransferase (*GlyE*, *EpsH*, *GlyG*) Capsular Protein cap8A	11	*EpsG* family protein Glycosyltransferase ABC transporter
	Bacteriocin	1	Lactacin-F subunit *LafA*	8	Helveticin-J
	Oxidative Response	4	Thioredoxin *YtpP* Thioredoxin reductase NADH peroxidase	6	DSBA-like thioredoxin Ferritin/DPS protein Flavin oxidoreductase *GrdB*-related oxidoreductase
Context-dependent	Protease, Peptidase	44	Aminopeptidase E Dipeptidase proteins Neutral endopeptidase Protease *HtpX*	16	CPBP metalloproteases CAAX proteases Endopeptidases *ImmA*/*IrrE* metallo-endopeptidase
	Stress Response	4	*RecA* Cold shock protein *CspA* Chaperone protein *DnaK* *LexA* repressor	1	Stress response proteins *GlsB*, *YeaQ*, *YmgE*

^a^ Prokka annotation of *L. paragasseri* HUH02 consensus assembly (3 contigs; total CDS: 2189; annotated: 1192). ^b^ Foldseek structural homology search (databases: pdb100, afdb50; 3Di + AA mode) against AlphaFold-predicted structures of 948 hypothetical proteins. Categorization based on best-hit descriptions (prob ≥ 0.9 = High confidence).

## Data Availability

The original data presented in the study are openly available in the NCBI Sequence Read Archive (SRA) under BioProject accession number PRJNA1469785. The data will be made publicly available upon publication of this article.

## Supplementary Materials

The following supporting information can be downloaded at https://www.mdpi.com/article/10.3390/microorganisms14081777/s1; Figure S1. Genome characteristics of *Lactobacillus paragasseri* HUH02. Circular representation of the *L. paragasseri* HUH02 genome generated with Proksee/CGView. Figure S2. Genome comparison of *Lactobacillus paragasseri* HUH02 and close strains. Comparative analysis performed in Orthovenn3 (https://orthovenn3.bioinfotoolkits.net, accessed on 24 July 2026). Figure S3. The phylogenetic tree of *Lactobacillus paragasseri* HUH02. Table S1. Safety assessment of *Lactobacillus paragasseri* HUH02 strain.

## Abbreviations

DPBS	Dulbecco’s phosphate-buffered saline
IL-1β	Interleukin-1 beta
IL-6	Interleukin-6
iNOS	Inducible nitric oxide synthase
LPS	Lipopolysaccharide
MITF	Microphthalmia-associated transcription factor
TNF-α	Tumor necrosis factor-alpha
TRP-1	Tyrosinase-related protein 1
TRP-2	Tyrosinase-related protein 2
TYR	Tyrosinase
ZO-1	Zonula occludens-1
