# Supplementary material for: Functional Characterization of Breast Milk-Derived Lactobacillus paragasseri HUH02 Postbiotic Preparations with Immunomodulatory, Skin Barrier-Protective, and Anti-Melanogenic Activities
Source: Microorganisms. 2026 Aug 12;14(8):1777. doi: 10.3390/microorganisms14081777 (PMC13515363; doi:10.3390/microorganisms14081777)
Supplement: Supplementary file 1 [file microorganisms-14-01777-s001.zip › microorganisms-4435904-supplementary.pdf]

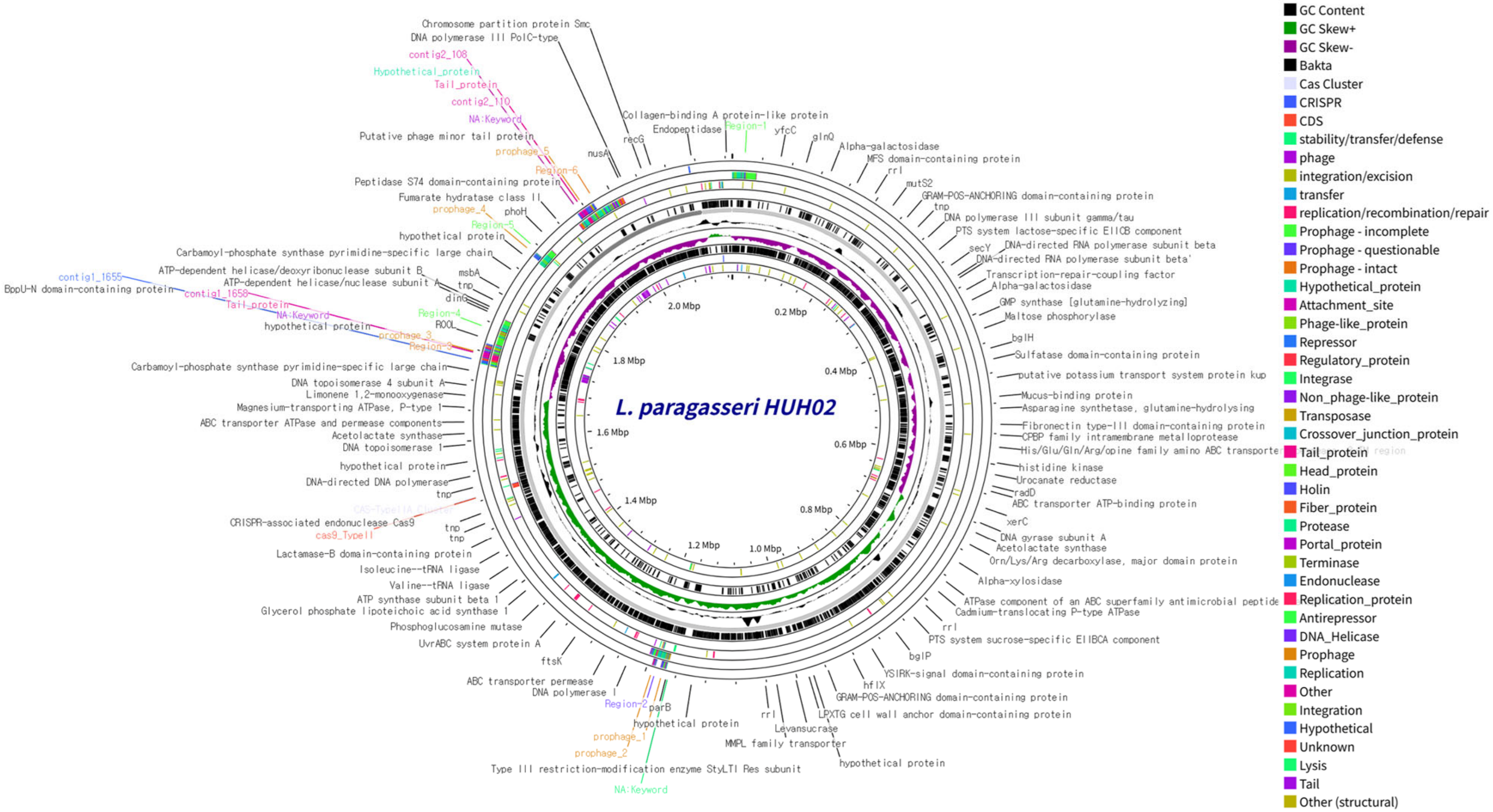

Figure S1. Genome characteristics of *Lactobacillus paragasseri* HUH02. Circular representation of the L. paragasseri HUH02 genome generated with Proksee/CGView.

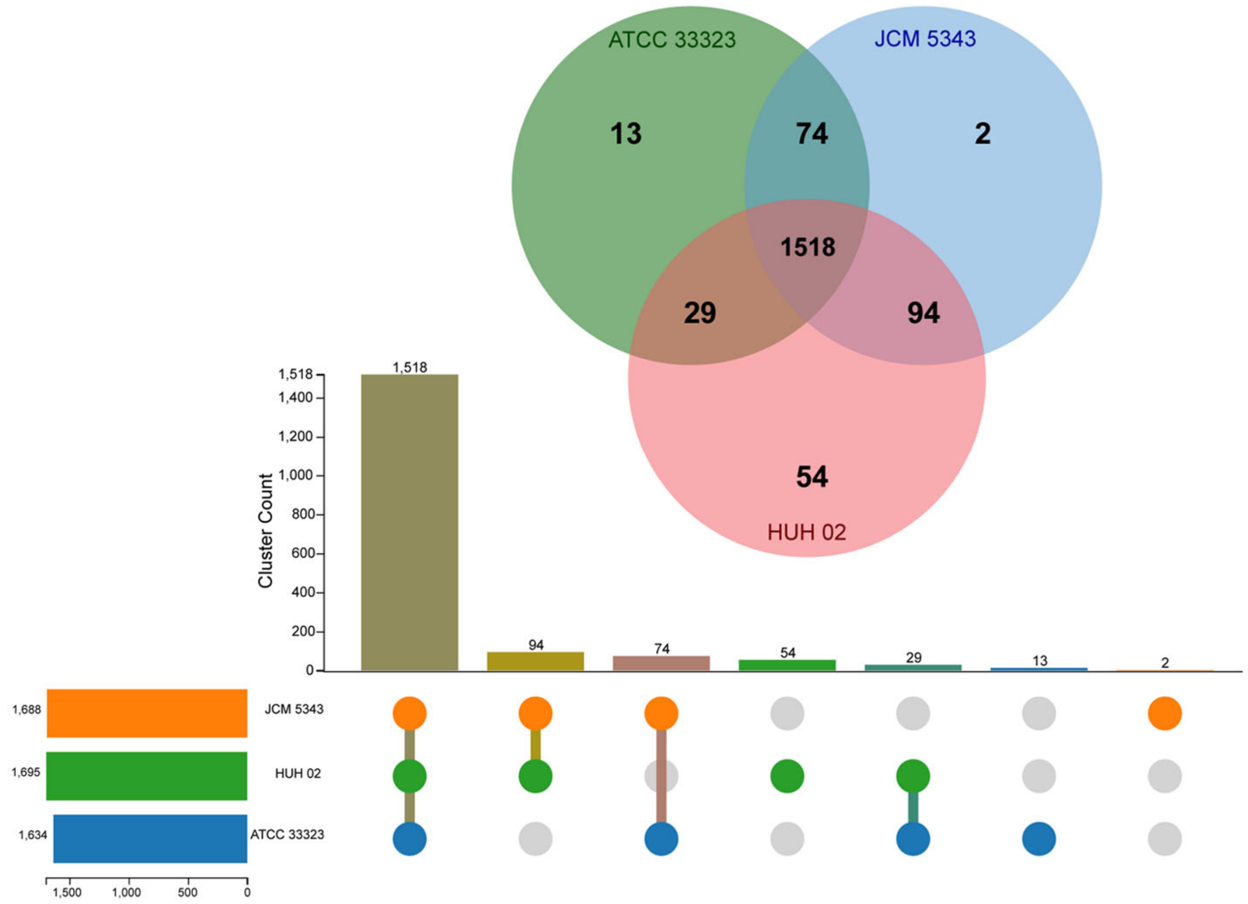

Figure S2. Genome comparison of *Lactobacillus paragasseri* HUH02 and close strains. Comparative analysis performed in Orthovenn3 (https://orthovenn3.bioinfotoolkits.net).

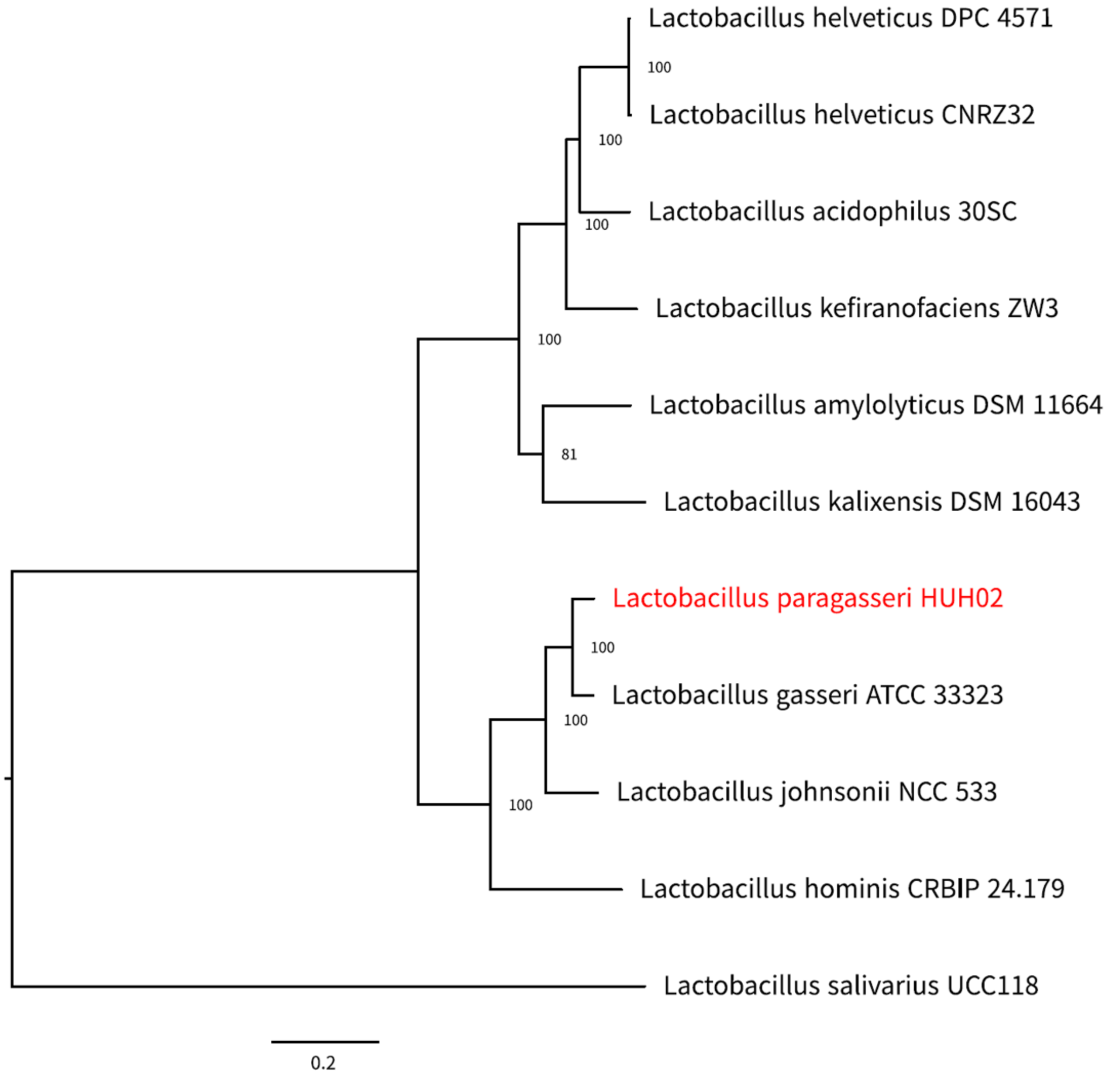

Figure S3. The phylogenetic tree of *Lactobacillus paragasseri* HUH02.

Table S1. Safety assessment of *Lactobacillus paragasseri* HUH02 strain.

	Minimum inhibitory concentrations (MICs) of antibiotics[a]									Gelatinase activity[c]	Hemolysis activity[c]
	AMP	CLI	CHL	ERY	GEN	KAN	STR	TET	VAN		
EFSA[b]	1	1	4	1	16	16	16	4	2	(-)	(-)
HUH02	0.047	0.032	1	<0.016	1	8	3	0.5	0.75	(-)	(-)

[a]MICs were expressed as the concentration μg/mL of antibiotics. AMP, ampicillin; CLI, clindamycin; CHL, chloramphenicol; ERY, erythromycin; GEN, gentamicin; KAN, kanamycin; STR, streptomycin; TET, tetracycline; VAN, vancomycin.

[b]EFSA (European Food Safety Authority) breakpoints were based on the EFSA FEEDAP Panel (2012). Guidance on the assessment of bacterial susceptibility to antimicrobials of human and veterinary importance: *Lactobacillus* obligate homofermentative.

[c]Positive control strain was *P. aeruginosa* PA01.
